# Acute invasive mucormycosis rhinosinusitis causing multigroup cranial nerve injury and meningitis—A case report

**DOI:** 10.3389/fneur.2022.873694

**Published:** 2022-10-04

**Authors:** Tingting Wang, Duanhua Cao, Jingzhe Han

**Affiliations:** Department of Neurology, Harrison International Peace Hospital, Hengshui, China

**Keywords:** Mucor, cranial nerve, 3D-SPACE technology, mNGS, infection

## Abstract

This study reported a case of a Rhino-Orbital-Cerebral Mycosis (ROCM) patient with multiple groups of cranial nerve damage as the primary clinical manifestation, confirmed by histopathology and cerebrospinal fluid metagenomic next-generation sequencing (mNGS) technology. Relying on the MRI3D-SPACE technology, we observed the location and extent of the cranial nerve damage in the patient. The results suggested that fungal meningoencephalitis caused by mucor may enter the skull retrograde along the cranial nerve perineurium. The patient was admitted to the hospital with a preliminary diagnosis of mucormycosis infection after 1.5 days of mouth deviation. We treated the patient immediately with intravenous amphotericin B liposomes. After 21 days of hospitalization, the clinical symptoms of the patient did not improve significantly. The patient was discharged due to financial difficulties and antifungal treatment at home, and his disease had stabilized at the 6-month follow-up.

## Introduction

Fungal infections of paranasal sinuses are common in the clinic and can be classified into invasive and noninvasive infections according to the progression of the disease (1, 2). Fungal infections of paranasal sinuses often require a differential diagnosis from chronic suppurative sinusitis, malignancy, sinus polyps, and necrotizing maxillary sinusitis (3–5). Early diagnosis of fungal sinusitis is usually critical, and most patients can be cured after early and timely diagnosis and reasonable treatment. Treatment is more complicated and prone to recurrence and poorer healing when delayed to a late stage (6, 7).

In 1969, Champion defined the simultaneous invasion of fungi into paranasal sinuses, eyes, and intracranial part of the brain as Rhino-Orbital-Cerebral Mycosis (ROCM), which belongs to acute invasive fungal rhinosinusitis (8). The main species responsible for invasive fungal rhinosinusitis infection are Mucor and Aspergillus, which belong to opportunistic pathogens (9, 10). The invasion routes can be divided into a direct spread and a transvascular invasion. Pathogens can enter the brain through the paranasal sinuses, orbit, extraocular muscles, ophthalmic artery, and optic nerve or drain to the cavernous sinus through the paranasal sinuses, orbital reflux veins, or intracranial infection. Studies found that patients with ROCM may present with multiple cranial nerve palsy (function of the cranial nerves II, III, IV, V, and VI may be lost or impaired) (11, 12). The magnetic resonance 3D-SPACE sequence is a variation of the TSE sequence. Through reconstruction, the complex anatomical structure of the cranial nerve can be further clearly displayed at any level and direction, which is vital for observing the path, distribution, and injury of the cranial nerve (13). In this study, we reported a ROCM patient with multiple groups of cranial nerve damage as the primary clinical manifestation, confirmed by histopathology and cerebrospinal fluid mNGS technology. We used the MRI3D-SPACE technique to provide detailed observation and better assess the extent of ROCM patients with cranial nerve injury due to the fungal meningoencephalitis caused by Mucor.

## Case description

**Figure 1:**
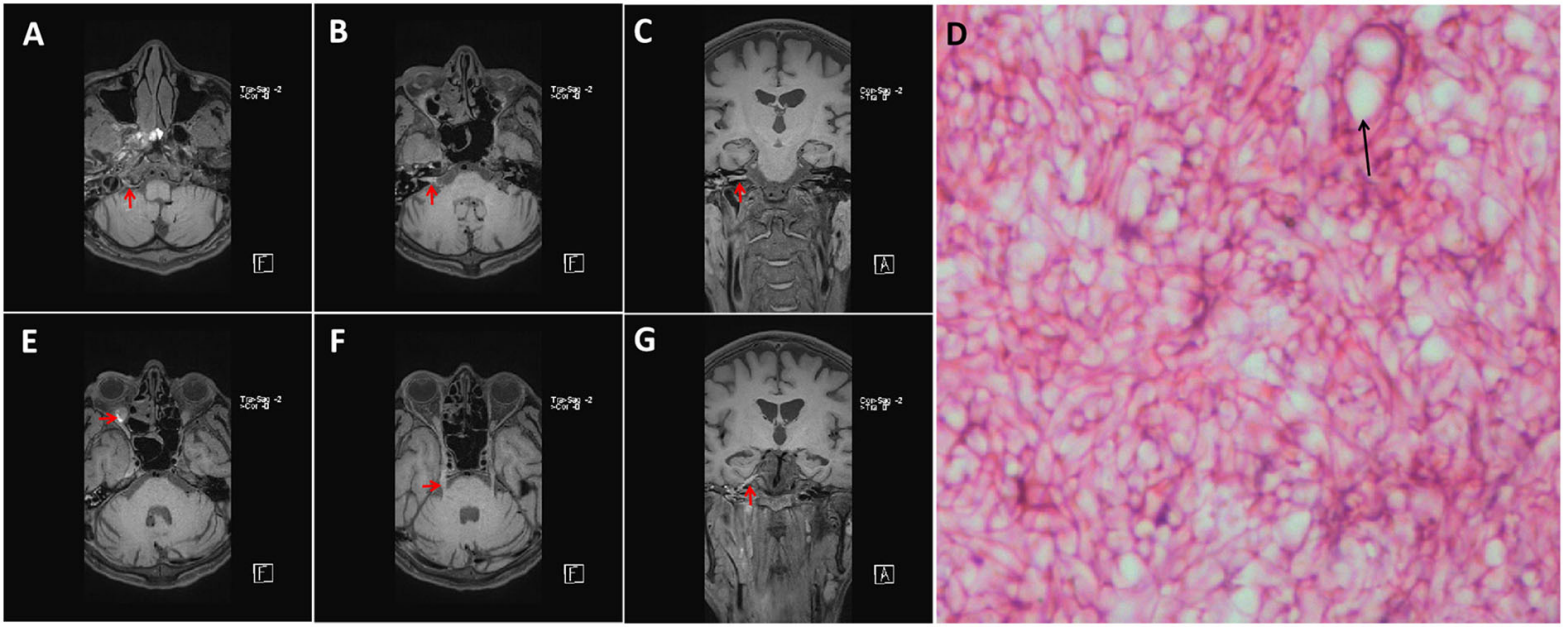
Enhanced MRI with 3D-T1-SPC sequence results of the patient (a standard dose of gadolinium). The MRI showed enhanced images of the right hypoglossal nerve **(A)**, the posterior orbital wall **(E)**, the facial nerve **(B,C)**, and the trigeminal nerve **(F,G)** slightly (Red Arrow). Microscopic photograph showing hyphae and Sporangium (Black Arrow) in HE staining [**(D)**, 400X].

A 36-year-old male patient was admitted to our hospital for mouth deviation for 1.5 days. The patient was admitted to the Department of Endocrinology 1 month ago due to diabetic ketoacidosis (10:00 a.m. on June 18, 2021). Physical examination showed that the patient had clear consciousness, less fluent speech, right peripheral facial paralysis, and a right deviation of the protruding tongue; his muscle strength and tension in four extremities were normal, and the bilateral Babinski sign was negative. Auxiliary urine routine examination revealed urine glucose of 4+, urine ketone body of 3+, urine protein of 1+, and glycosylated hemoglobin of 10.1%. No apparent abnormalities were observed on cranial MRI. Chest CT showed small solid nodules in the upper lobe of the right lung and the lower lobe of the left lung; ground-glass opacities in the medial segment of the right middle lobe indicated some inflammatory changes. Abdominal CT showed many colonic contents, which should be diagnosed based on clinical symptoms and regarded as minor bowel dysfunction. At admission, the random fingertip blood glucose and blood ketones were 20.1 mmol/L and 6.4 mmol/L, respectively. After admission, the patient's condition gradually worsened, with restricted right eye movement, hoarseness, occasional febrile headache, nausea, and vomiting. On the fourth day of admission, the patient had clear consciousness, a hoarse voice, right eyelid ptosis, and eye proptosis, which was fixed in the primary position. The patient's right frontal striae disappeared, the right nasolabial fold was shallow, and his tongue was extended to the right. The patient had hypoalgesia on the right side of the face, normal muscle strength in the extremities, and meningeal stimulation signs (+). The complete set of immune indexes and five preoperative indicators were normal. The lumbar puncture indicated the pressure of 150 mmH_2_O, a yellowish color cerebrospinal fluid, white blood cells of 109 × 10^6^/L (0–8 × 10^6^/L), protein of 1,003 mg/L (0.15–0.45 g/L), chloride of 111.2 mmol/L, and glucose of 5.54 mmol/L. Cerebrospinal fluid cytology tests showed a mixed cellular response. mNGS of *Rhizopus oryzae*'s sequence number was 19 (Total length was 1350 bp, 0.00334% coverage, and 1.00 X average depth). Repeated head MRI showed the inflammation of cerebellar infarction, right paranasal sinuses, and ethmoid sinuses. The plain and enhanced 3D-SPACE sequence scanning showed abnormal enhancement of the right hypoglossal nerve, the facial nerve, the cisternal segment of the trigeminal nerve, the posterior wall of the orbit, the periphery of the right maxillary sinus, and the oral maxillofacial spaces (Figure 1). Amphotericin B liposome (triamcinolone acetonide) was immediately intravenously administered at an initial dose of 5 mg/d, and no significant side effects were observed. Then, 5 mg of amphotericin B was added daily until the maintenance dose of 40 mg/day was reached. On the seventh day of admission, the specimen of the patient was taken for pathological examination with the assistance of the otorhinolaryngology and the Department of Stomatology. After 1 week, the patient was diagnosed with mucormycosis infection due to microscopic visualization of Molds hyphae (Figure 1). After 21 days of hospitalization, the clinical symptoms of the patient did not improve significantly. The patient was discharged due to financial difficulties and was provided antifungal treatment at home. During hospitalization, the patient was on antifungal medication (liposomal amphotericin B) for 17 days; after discharge, he was switched to oral antifungal (voriconazole) for 6 months. During the six-month follow-up, the condition of the patient was relatively stable.

## Discussion

Mucor belongs to the class Zygomycetes, order Mucorales, and the most common pathogenic bacteria are Rhizopus, Mucor, and Oryzae in the family Mucoraceae (14). In this case, the patient was infected with *R. oryzae*. The patient had been admitted to the Department of Endocrinology due to diabetic ketoacidosis. Mucormycosis has a complex disease course, rapid progression, high mortality, and intricate clinical manifestations. It is divided into six types, of which the naso-orbital brain type accounts for 36.4%. This patient was finally diagnosed with ROCM when combined with the clinical characteristics of sinus, orbit, meninges, cranial nerve injury, and pathogen detection. The case was complicated due to the involvement of the cranial nerve, meninges, and infarction in the distribution area of the posterior inferior cerebellar artery, suggesting that both pathways were involved in the pathogenic process.

Studies showed that the prevalence of fungal sinus disease has been increasing in recent decades. This may be due to increased awareness of medical examinations, overuse of antibiotics, and increased use of immunosuppressive drugs (15). Common clinical manifestations of ROCM include headache, fever, black nasal eschar, orbitofacial cellulitis, cranial nerve palsy, altered sensorium, and hemiparesis (16). However, in the present study, the patient was first admitted to the neurology department with a cranial nerve injury. His disease gradually aggravated to multiple groups of cranial nerve damage, leading to meningitis-like features that appeared successively. Early diagnosis is difficult because the patient has no nasal infection and other symptoms such as nasal congestion, runny nose, epistaxis, and typical ROCM imaging manifestations. Cranial nerves reach various parts of the head and the neck through the bony foramen of the skull base and meninges; hence, they are vulnerable to multiple local or systemic lesions throughout the process. Fungal infections such as mucormycosis can affect numerous cranial nerves and lead to fungal meningitis. ROCM is the most common clinical form of mucormycosis infection. Mucor can be transmitted *via* the nasal cavity, the paranasal sinuses, thje neck, and the orbit into the cranial cavity, causing brain infection and involvement of the cranial nerves (17). The definite diagnosis and treatment include a comprehensive analysis of the patient, finding the site of cranial nerve injury, and performing a lumbar puncture to find pathogens. Due to the slender anatomy, complex course, and overlapping surrounding structures of cranial nerves, standard radiographic methods cannot show their structures well. With the advancement of technology, 3D high-resolution body scanning technology can display the cranial nerve structure through 3D reconstruction and accurately measure each cranial nerve's course angle and brain cisternal segment length (13). The 3D-SPACE sequence has the best imaging effect on cranial nerves (especially the cisternal segment), and the scanning time is shorter. 3D-SPACE sequence enhancement showed abnormal enhancement of the right hypoglossal nerve, the facial nerve, and the trigeminal nerve's cisternal segment, which revealed the degree and location of cranial nerve injury. In addition, it was also found that the enhancement of cranial nerve was more pronounced at sites distant from the brainstem; no damage was found at the junction of cranial nerves and brainstem. The above two studies suggested that trichinosis can enter the skull retrogradely along the cranial nerves, adding to the etiological theory of intracranial infection. At the same time, relying on 3D-SPACE technology, it was also possible to assess the degree of mucormycosis cranial invasion and the effect of antifungal therapy. However, further studies with a large specimen size are still needed. The right eyeball of the patient was fixed without exophthalmos, and the possibility of incomplete orbital apex syndrome or superior orbital fissure syndrome was considered. The enhancement of the posterior wall of the eyeball could explain this clinical manifestation. Studies found that the appearance of orbital apex syndrome is highly suggestive of fungal infection, and the symptoms at this stage are typical of ROCM (18).

Cerebrospinal fluid testing showed that the patient was experiencing an inflammatory response. Therefore, screening for pathogens, in this case, becomes a critical adjunctive test to confirm the diagnosis of ROCM. An mNGS examination was performed directly to find the pathogen to confirm the diagnosis further. The mNGS technology has significant advantages in pathogen screening (19, 20), especially when suspected pathogen species are not identified. The patient was rapidly confirmed to have *R. oryzae* infection by mNGS and was given antifungal therapy. The diagnosis of ROCM in the patient was confirmed by analyzing the source of Mucor, which was finally approved by histopathology. If patients with ROCM can be diagnosed early, they can receive active treatment. However, the rarity of ROCM leads to a high rate of misdiagnosis. Many cases are diagnosed before the end of life or even after the autopsy, so the mortality rate is also very high. Therefore, early lumbar puncture and rapid mNGS detection may indicate a better prognosis in patients with suspected fungal meningitis.

## Conclusion

ROCM is easily misdiagnosed clinically and patients suffering from ROCM are susceptible to disability and death without timely treatment. In patients with a high clinical suspicion of ROCM, an MRI 3D-SPACE sequence is required to assess the extent of cranial nerve injury and to determine whether the pathogen has entered the skull. Cerebrospinal fluid mNGS technology offers tremendous advantages in pathogen screening. In patients with ROCM, signs of retrograde entry along cranial nerves and invasion of the meninges or brainstem should be promptly screened for pathogens from imaging by using cerebrospinal fluid mNGS technology. Fluconazole, itraconazole, and caspofungin are preferred for the prophylactic treatment of fungal infections. However, amphotericin B is widely accepted as an effective drug for treating Trichophyton. Its clinical application is limited due to its tendency to cause severe hepatic and renal side effects. Liposomal amphotericin B can be used preferentially in patients with ROCM because it has fewer therapeutic side effects than both amphotericin B. Besides, early aggressive and extensive surgical excision of fungal-infected inactivated tissue is also a preferred option. Early diagnosis and treatment are decisive for improving the prognosis of ROCM, and magnetic resonance 3D-SPACE sequence combined with mNGS technology may be of clinical application for early diagnosis of ROCM.

## Data availability statement

The raw data supporting the conclusions of this article will be made available by the authors, without undue reservation.

## Ethics statement

The studies involving human participants were reviewed and approved by the Ethics Committee of Harrison International Peace Hospital. The patients/participants provided their written informed consent to participate in this study. Written informed consent was obtained from the individual(s) for the publication of any potentially identifiable images or data included in this article.

## Author contributions

JH and TW organized and proofread the writing of the editorial. DC and TW wrote the manuscript draft. All authors contributed to the article and approved the submitted version.

## Conflict of interest

The authors declare that the research was conducted in the absence of any commercial or financial relationships that could be construed as a potential conflict of interest.

## Publisher's note

All claims expressed in this article are solely those of the authors and do not necessarily represent those of their affiliated organizations, or those of the publisher, the editors and the reviewers. Any product that may be evaluated in this article, or claim that may be made by its manufacturer, is not guaranteed or endorsed by the publisher.

## References

[1] Nourizadeh N Adabizadeh A Zarrinfar H Majidi M Jafarian AH Najafzadeh MJ. Fungal biofilms in sinonasal polyposis: the role of fungal agents is notable? J Oral Maxillofac Pathol. (2019) 31:295–8. 10.1016/j.ajoms.2019.01.007

[2] Bakhshizadeh M Hashemian HR Najafzadeh MJ Dolatabadi S Zarrinfar H. First report of rhinosinusitis caused by neoscytalidium dimidiatum in Iran. J Med Microbiol. (2014) 63:1017–9. 10.1099/jmm.0.065292-0 24850881

[3] Sakci Z Aydin F Ceylan O Ogul H. Sinonasal teratocarcinosarcoma mimicking chronic invasive fungal disease of paranasal sinuses. Ann R Coll Surg Engl. (2021) 103:e193–e5. 10.1308/rcsann.2020.7088 33852367 PMC10334927

[4] Li Z Wang X Jiang H Qu X Wang C Chen X. Chronic invasive fungal rhinosinusitis vs sinonasal squamous cell carcinoma: the differentiating value of MRI. Eur Radiol. (2020) 30:4466–74. 10.1007/s00330-020-06838-1 32279114

[5] Chen X Xian J Lu X. Value of Ct in the differential diagnosis of pseudomonas aeruginosa sinusitis and fungal ball in paranasal sinus caused by aspergilus flavus. Zhonghua yi xue za zhi. (2019) 99:3417–9. 10.3760/cma.j.issn.0376-2491.2019.43.013 31752470

[6] Dykewicz MS Rodrigues JM Slavin RG. Allergic fungal rhinosinusitis. J Allergy Clin Immunol. (2018) 142:341–51. 10.1016/j.jaci.2018.06.023 30080526

[7] Zhou M Zhao Y Zhang Y Zhang S. Clinical analysis of combined treatment in 87 patients with recurrent allergic fungal rhinosinusitis. Lin Chuang er bi yan hou tou Jing wai ke za zhi. (2018) 32:541–4. 10.13201/j.issn.1001-1781.2018.07.016 29798088

[8] Champion C Johnson T. Rhino-orbital-cerebral phycomycosis. Wien med News. (1969) 68:807–10. 5798251

[9] Hosseinikargar N Basiri R Asadzadeh M Najafzadeh MJ Zarrinfar H. First report of invasive aspergillus rhinosinusitis in a critically ill Covid-19 patient affected by acute myeloid leukemia, Northeastern Iran. Clini Case Rep. (2021) 9. 10.1002/ccr3.4889 34631073 PMC8489390

[10] Dolatabadi S Ahmadi B Rezaei-Matehkolaei A Zarrinfar H Skiada A Mirhendi H. Mucormycosis in Iran: a six-year retrospective experience. J Mycol Med. (2018) 28:269–73. 10.1016/j.mycmed.2018.02.014 29545123

[11] Song Y-M Shin SY. Bilateral ophthalmic artery occlusion in rhino-orbito-cerebral mucormycosis. Korean J Ophthalmol. (2008) 22:66–9. 10.3341/kjo.2008.22.1.66 18323710 PMC2629957

[12] Singh N Garg S Kumar S Gulati S. Multiple cranial nerve palsies associated with type 2 diabetes mellitus. Singapore Med J. (2006)47:712. 16865214

[13] Wu W Wu F Liu D Zheng C Kong X Shu S. Visualization of the morphology and pathology of the peripheral branches of the cranial nerves using three-dimensional high-resolution high-contrast magnetic resonance neurography. Eur J Radiol. (2020) 132:109137. 10.1016/j.ejrad.2020.109137 33022550

[14] Khor B-S Lee M-H Leu H-S Liu J-W. Rhinocerebral mucormycosis in Taiwan. J Microbiol Immunol Infect. (2003) 36:266–9. 14723256

[15] Deutsch PG Whittaker J Prasad S. Invasive and non-invasive fungal rhinosinusitis—a review and update of the evidence. Medicina. (2019) 55:319. 10.3390/medicina55070319 31261788 PMC6681352

[16] Nussbaum ES Hall WA. Rhinocerebral mucormycosis: changing patterns of disease. Surg Neurol. (1994) 41:152. 10.1016/0090-3019(94)90114-7 8115954

[17] Vivek P Rima S Ritesh K Chaitanya BS Bhujang P. Rhino-orbito-cerebral mucormycosis: pictorial review. Insights Imag. (2021) 12. 10.1186/s13244-021-01109-z 34767092 PMC8587501

[18] Arndt S Aschendorff A Echternach M Daemmrich TD Maier W. Rhino-orbital-cerebral mucormycosis and aspergillosis: differential diagnosis and treatment. Eur Arch Oto-Rhino-L. (2009) 266:71–6. 10.1007/s00405-008-0692-y 18470529

[19] Wilson MR Naccache SN Samayoa E Biagtan M Bashir H Yu G. Actionable diagnosis of neuroleptospirosis by next-generation sequencing. N Engl J Med. (2014) 370:2408–17. 10.1056/NEJMoa1401268 24896819 PMC4134948

[20] Guan H Shen A Lv X Yang X Ren H Zhao Y. Detection of virus in CSF from the cases with meningoencephalitis by next-generation sequencing. J Neurovirol. (2016) 22:240–5. 10.1007/s13365-015-0390-7 26506841

